# Investigation of the Mechanical and Electrical Properties of Elastic Textile/Polymer Composites for Stretchable Electronics at Quasi-Static or Cyclic Mechanical Loads

**DOI:** 10.3390/ma12213599

**Published:** 2019-11-01

**Authors:** Christian Dils, Lukas Werft, Hans Walter, Michael Zwanzig, Malte von Krshiwoblozki, Martin Schneider-Ramelow

**Affiliations:** 1 Fraunhofer IZM (Institute for Reliability and Microintegration), 13355 Berlin, Germany; Lukas.Werft@izm.fraunhofer.de (L.W.); Hans.Walter@izm.fraunhofer.de (H.W.); Michael.Zwanzig@izm.fraunhofer.de (M.Z.); Malte.von.Krshiwoblozki@izm.fraunhofer.de (M.v.K.); 2 Microperipheric Center, Technical University Berlin, 10623 Berlin, Germany; Martin.Schneider-Ramelow@izm.fraunhofer.de

**Keywords:** textile/polymer composite, stretchable electronics, smart textiles, mechanical and electrical properties, quasi-static and cyclic mechanical loading, life-time expectancy

## Abstract

In the last decade, interest in stretchable electronic systems that can be bent or shaped three-dimensionally has increased. The application of these systems is that they differentiate between two states and derive there from the requirements for the materials used: once formed, but static or permanently flexible. For this purpose, new materials that exceed the limited mechanical properties of thin metal layers as the typical printed circuit board conductor materials have recently gained the interest of research. In this work, novel electrically conductive textiles were used as conductor materials for stretchable circuit boards. Three different fabrics (woven, knitted and nonwoven) made of silver-plated polyamide fibers were investigated for their mechanical and electrical behavior under quasi-static and cyclic mechanical loads with simultaneous monitoring of the electrical resistance. Thereto, the electrically conductive textiles were embedded into a thermoplastic polyurethane dielectric matrix and structured by laser cutting into stretchable conductors. Based on the characterization of the mechanical and electrical material behavior, a life expectancy was derived. The results are compared with previously investigated stretchable circuit boards based on thermoplastic elastomer and meander-shaped conductor tracks made of copper foils. The microstructural changes in the material caused by the applied mechanical loads were analyzed and are discussed in detail to provide a deep understanding of failure mechanisms.

## 1. Introduction

Stretchable circuit boards are required for applications where an applied single or repeated mechanical load results in deformation of the system without loss of the desired functionality. In the first case, electronic components are integrated onto two-dimensional substrates that are easier to manufacture than complex three-dimensional ones, before the final shaping. In these forming processes, one-time loads act on the circuit carriers [1]. In the second case, electronics are integrated into systems that are repeatedly stretched. These include, for example, smart clothing for safety, work, sport or healthcare applications, conductors in soft robotics, and wearable sensors in medical bandages or skin-adhesive patches. All applications require non-destructive single or multiple deformation of the circuit board [2]. Current commercial solutions are based on a non-linear design of copper-based conductor tracks, with limited mechanical reliability. In this work, therefore, the copper foil-based conductors are substituted with conductive fabrics.

As is known, commonly used textile fibers show no intrinsic electrically conductive properties. However, the raw fiber textile can be processed into an electrically conductive material by plating a thin metal layer onto their surface, in which gold, silver or copper is usually applied [3]. These fibers are further twisted into electrically conductive yarns that can be embroidered into conductive tracks and even used to contact electronic modules [4]. Tensile tests have shown that such metallized yarns have an elongation at a break of 20%, with the initial electrical resistance quadrupling before breakage [5]. It has also been reported that a thin silver plating has no influence on the tear strength of the fiber; however, the thermoelectric properties of conductive filaments should be considered for the respective processing and application [6]. Conductor tracks based on metallized yarns can be stretched to nearly 50% by embroidering them as a zigzag structure onto elastic fabrics [7]. Yet, in wearable applications, elastic loads can occur from 5% (back) to 60% (along the elbow). Embroidered conductive yarns therefore do not meet the mechanical requirements for reliable use, especially for tight, body-worn garments that are used to measure vital signs and are therefore subject to high dynamic mechanical loads. Even elastic fabric-embedded and preconditioned stainless steel filaments do not exhibit sufficient mechanical and electrical properties under cyclic loading [8].

**Figure 1:**
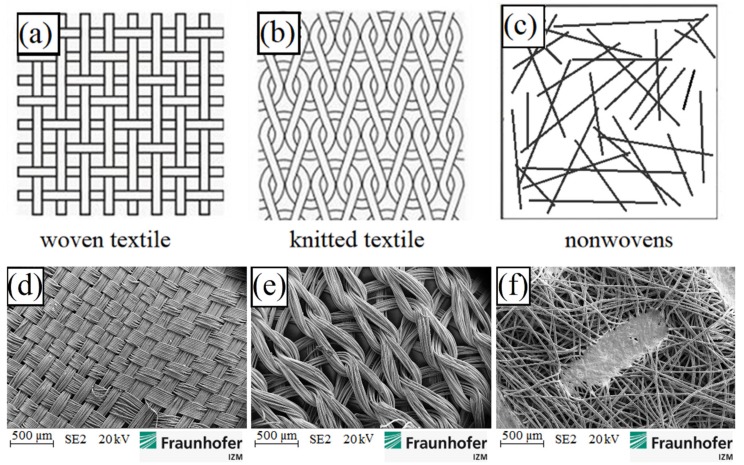
Schematic drawings above of: (**a**) woven; (**b**) knitted and (**c**) nonwoven fabrics and SEM images below of: (**d**) woven; (**e**) knitted and (**f**) nonwoven fabrics.

Thus, we propose the use of metallized polymer-based fabrics, which, due to textile processing techniques, produce a material with higher ductility than a single yarn- or fabric-based material on metal wires. The investigation of the mechanical and electrical properties of these materials, structured into conductor tracks and embedded in an elastic matrix, is the object of this article. In Figure 1, the schematic drawings and SEM images of three different types of selected conductive fabrics are shown.

## 2. Materials and Methods 

All selected fabrics are made of silver-plated polyamide (PA 6.6) fibers, which are embedded in thermoplastic polyurethane (TPU). Important material properties are summarized in Table 1.

### 2.1. Thermoplastic Polyurethane (TPU)

Thermoplastic polyurethane in film form is used as a substrate material. TPU combines the properties of thermoplastics and elastomers. At temperatures above the glass transition temperature (at −40 °C), TPU has an elastic behavior. This feature is required for later application as a stretchable circuit board. In contrast to elastomers, the selected TPU film also has a melting point (at 160 °C). Above this temperature, it can be thermoformed [13].

### 2.2. Polyamide 6.6 (PA 6.6)

In the textiles industry, polyamide is the second most used man-made fiber [14]. It is a flexible, thermoplastic polymer that is characterized by high strength. Polyamide filaments (single fibers) are made by melt spinning [14]. The molten polymer is pressed through spin dosage plates, frozen by cooling it down under airflow, and extended to a multiple of its original length. While stretching, the crystalline areas in the polyamide slide off each other. Thereby, they are aligned in the pulling direction. The polyamide becomes stronger and stiffer and the elasticity decreases. The stretching of the polyamide can be partially undone by a heat treatment, at which the required temperature is between the glass transition temperature and the melting temperature. The crystalline areas take on a more energetically favorable form. Under mechanical load, this tempered polyamide filament is more elastic than the non-tempered one [9].

### 2.3. Thin Silver Layer (On PA 6.6 Filaments)

The fabrics, made from polyamide fibers, are plated with a thin silver layer. Silver is chosen because of its high electrical conductivity and chemical resistance [15]. In addition, Krshiwoblozki [16] described silver as a preferred surface metallization for contacting electronic modules on textile conductors by means of a non-conductive adhesive bonding process. The silver layer on the selected fabrics is around 50–200 nm thick and consists of many silver crystals smaller than 100 nm. It is not a uniform layer as it is known from foil.

### 2.4. Manufacturing of Elastic Textile/Polymer Composites for Stretchable Circuit Boards

**Figure 2:**
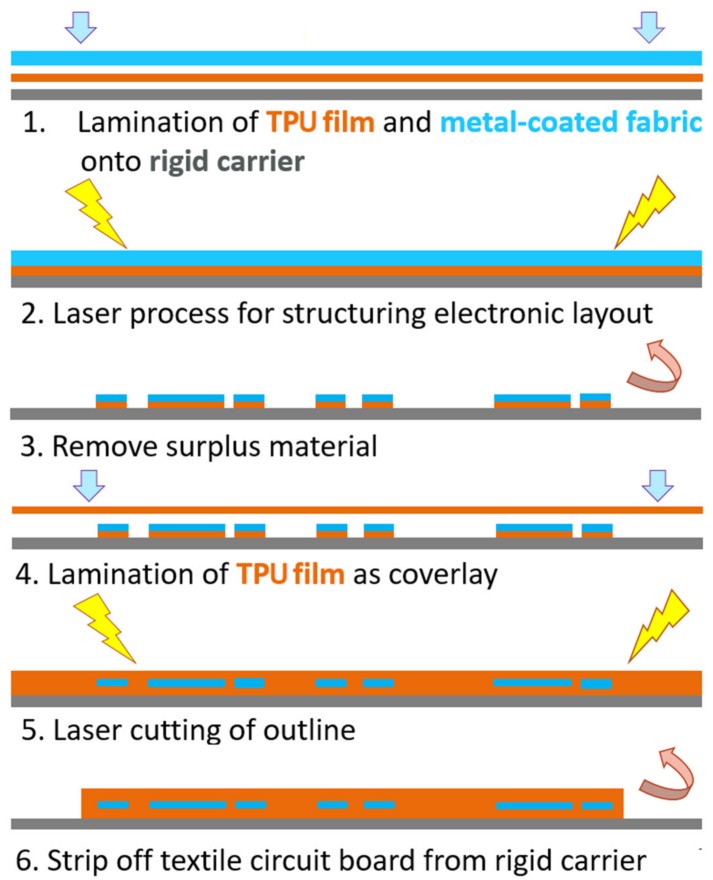
Process flow of stretchable circuit boards based on elastic textile/polymer composites.

For electrical insulation and protection against external agents, the conductor tracks are embedded in a matrix of TPU. Figure 2 illustrates the developed process flow in the manufacturing of stretchable circuit boards made of textiles and elastomer films.

**Figure 3:**
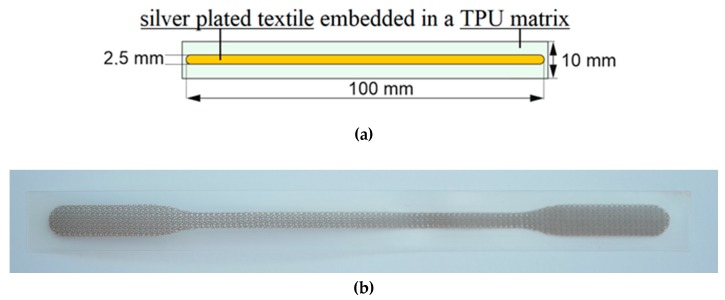
Design of test piece: (**a**) schematic design; (**b**) realized test sample.

A rigid carrier is used to improve handling during manufacturing. A TPU film and a conductive fabric are fixed and bonded at an elevated temperature and pressure onto the carrier (see Figure 2, step 1). The electrical conductor track is structured using a CO_2_-laser (see step 2). The excess material is removed, so only the structure of the conductive pattern remains on the carrier (see step 3). Afterwards, a second TPU film is laminated as a cover layer (see step 4). Then the sample outline is laser-cut. Finally, the stretchable composite can be lifted from the rigid carrier (see steps 5 and 6). In Figure 3 the design and the realized test pieces are shown.

It has been observed that the lamination of conductive fabrics has a significant impact on the electrical and mechanical properties of the material. In general, the metallized polyamide filaments are contacted to each other selectively, not over their entire surface. The number of contact points per surface again differs between the woven, the knitted and nonwoven fabric, depending on the voids and type of the thread intersections. Depending on the textile processing, the resulting contact pressure between the filaments plays an important role in the electrical resistance according to Holm’s contact theory [17]. Furthermore, the silver on the PA 6.6 filaments does not consist of a continuous layer. Rather, it is made up of many tiny silver nanoparticles. The silver-plating has an uneven thickness of 50 nm to 200 nm, so the electrical conductivity is limited by the thinnest area.

**Figure 4:**
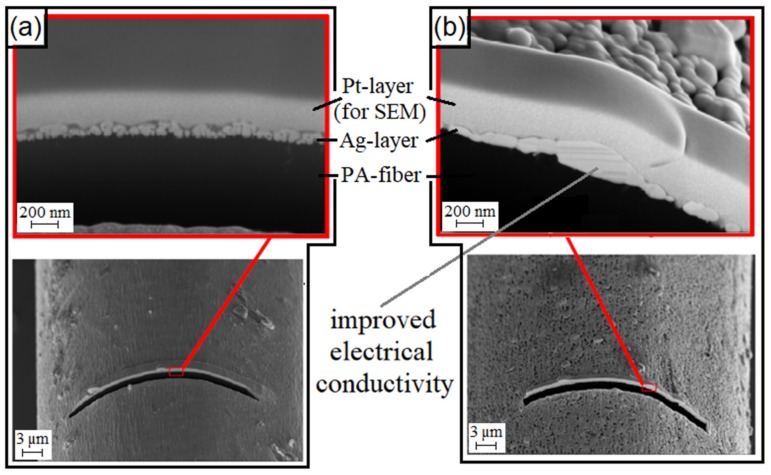
SEM-image of a cut with a focused ion beam (FIB) on a thermally: (**a**) untreated; (**b**) tempered silver-plated PA 6.6 [18].

Temperatures far below the melting temperature of silver, which also occur while laminating the samples, have a great influence on the silver layer. The individual silver nanoparticles sinter together to form a more compact layer (see Figure 4), whereby the contact area is increased, and the electrical conductivity is improved.

**Figure 5:**
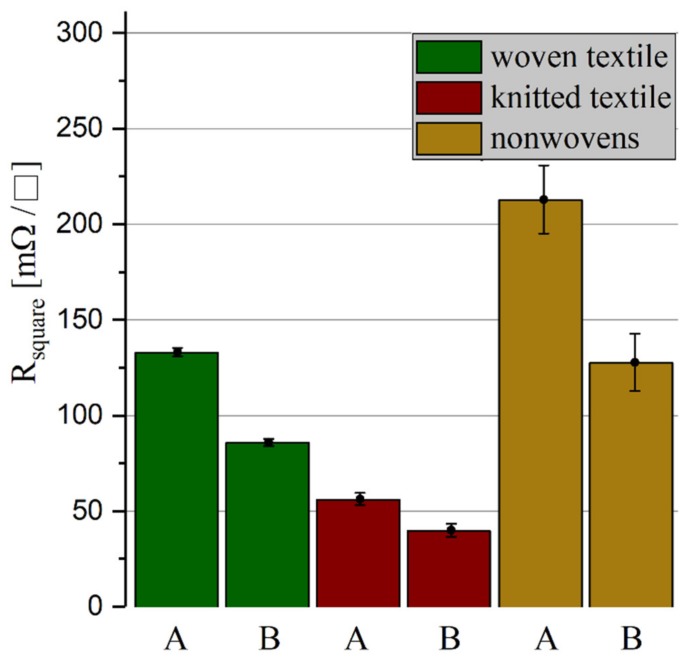
Sheet resistances of the three different fabrics before (**A**) and after (**B**) the influence of pressure and heat during lamination.

The sheet resistance is used to compare the electrical resistances of two-dimensional conductors with different width and lengths, such as electrically conductive fabrics. It is calculated from the measured resistance and the quotient of conductor width and length ($R_{\;square}=R\;\cdot \frac{width}{length})$. The sheet resistances of the individual fabrics in different stages of processing are shown in Figure 5. The starting sheet resistances of the textile structures before lamination (without heat and pressure influence, marked with A) are compared to the resistances of the textile structures after lamination (with heat and pressure influence, for sheet resistance measurement without embedding in TPU, marked with B).

The test pattern with conductor tracks made of knitted textiles shows the lowest electrical resistance of the three tested fabrics, followed by the woven and the nonwoven structure. In all fabrics, the electrical resistance has dropped by about one third due to the lamination.

### 2.5. Quasi-Static Mechanical Load

For simulating the quasi-static mechanical load to the samples, the material testing machine TIRAtest 28025 (TIRA GmbH, Schalkau, Germany) is used for the uniaxial tensile test. Simultaneously, the electrical resistance of the conductor tracks is measured. For this purpose, the test samples are contacted with crimp connectors and connected to a digital multimeter Fluke 175 (Fluke Corporation, Everett, WA, USA). The boundary conditions for these tests are listed in Table 2.

### 2.6. Cyclic Mechanical Load

**Figure 6:**
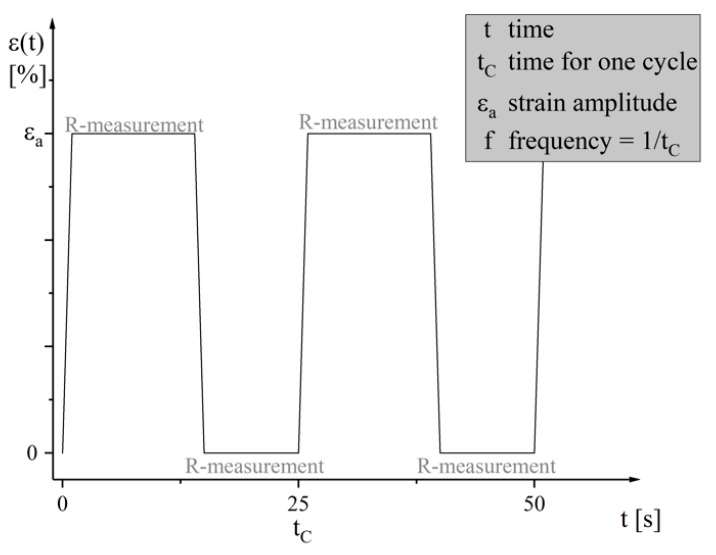
Time-progress of the strain-controlled cyclic tests.

The cyclic mechanical load, which acts on the stretchable circuit boards in many dynamic applications, is simulated by repeated uniaxial loading and relaxation. In order to characterize the change of the electrical behavior during the cyclic mechanical load test, the stretch tester developed and described by Born [19] is used. This measures the initial resistance of the clamped samples, pulls them to a preset stretch, further measures the electrical resistance in the stretched state, and then returns to the home position to measure the electrical resistance again. The stretch tester repeats these cycles until the conductor breaks or the test is terminated manually. The boundary conditions during these cyclic mechanical load tests are listed in Table 3 and Figure 6, the time-progress of the strain-controlled cyclic tests is shown.

## 3. Results

### 3.1. Quasi-Static Mechanical Load

**Figure 7:**
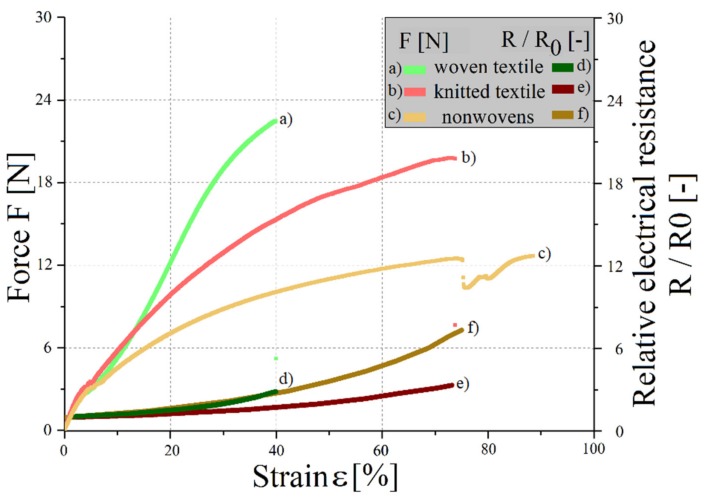
Force-strain diagram and relative electrical resistance changes at a quasi-static load.

The results of the tested mechanical and electrical properties of the three elastic textile/polymer composite samples are listed in Table 4 and shown in Figure 7. The following formula is used to calculate the initial sheet resistance in Table 4:

(1) $$R_{0,\;square}=R_{0}\cdot \frac{b}{l}=R_{0}\cdot \frac{2.5\;mm}{100\;mm}=\frac{R_{0}}{40}$$

In the case of woven and knitted structures, the mechanical damage causes the electrical failure. When the elastic textile/polymer composite breaks at about 38% and 72%, respectively, the silver structure also breaks. For nonwovens, the loosely connected filaments are pulled apart. Only a small amount of mechanical load acts on the fabrics. From an elongation of approx. 75%, the sample is no longer electrically conductive, as the filaments have completely separated from each other. Furthermore, the TPU matrix holds the composite together. The samples with conductor tracks made of nonwovens have a high initial resistance with great fluctuations. While quasi-static mechanical loading, they show the highest relative increase in resistance of the investigated structures. Due to their inconstant mechanical and electrical properties, the nonwoven fabrics enable no reliable use of them, so consequently they are excluded from further investigations. The knitted fabric shows the lowest initial and breaking resistance, as well as the lowest relative increase in resistance under quasi-static mechanical load. Likewise, it has the highest elongation at break of the tested structures. It is, regarding the mechanical and electrical properties, the most suitable structure for single forming.

**Figure 8:**
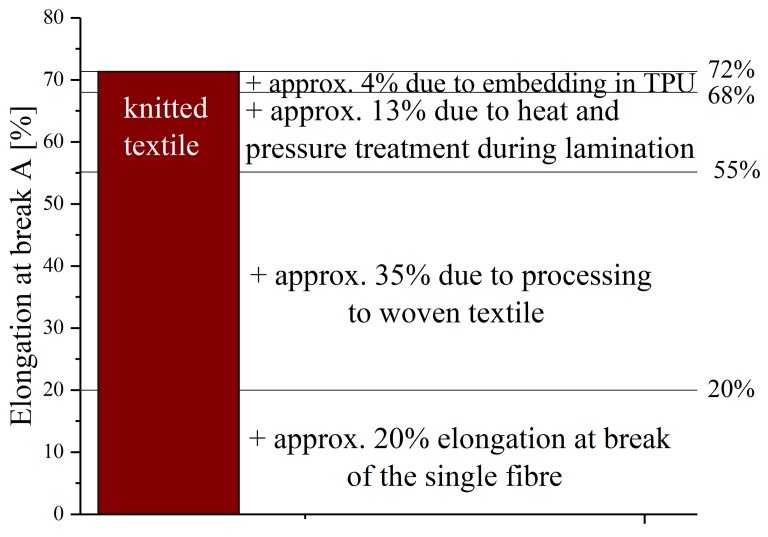
Influence of the process steps on the elongation at break point of the knitted fabric samples (absolute indication of the elongation at break point).

As expected, the elongation at break of the threads (A = 20%, [5]) is lower than those of the textile structures. The increased stretchability is attributable to, inter alia, the processing into fabrics. The tensile load of the fabrics is partly modified by the fact that the thread intersections strengthen under bending loads, so that a higher elasticity is achieved. In addition to processing into fabrics, the threads are also subjected to heat and pressure during lamination in TPU. In order to find out what influences the individual process steps have on the samples, further tests are conducted. Figure 8 illustrates the elongation at break point of all steps during processing, exemplary for samples made of knitted textiles.

The stretchable circuit boards have an average elongation at break point of about 72%. Knitted fabric samples that have undergone the same temperature and pressure conditions as the processed circuit boards, but which have not been embedded in TPU, have an elongation at break point of 68%. The non-tempered knit fabric is stretchable up to 55%. The increase in stretchability by heat and pressure treatment is due to a reduction of the degree of stretching (compare to Section 2.2). This leads to reduced strength and increased ductility.

All in all, it can be concluded that the processing of the threads into textiles increases the elongation at break point of the knitted structure by 35% (in absolute percentage). An equivalent evaluation of the results has shown that processing the fibers to woven structures increases the stretchability only by 2%. With 38% (woven-) to 72% (knitted fabric), the stretchable conductors made of textile structures have a higher elongation at break point than the silver-plated polyamide yarns (A = 20%, [5]). Other approaches to stretchable circuit boards, such as those with meander-shaped tracks made of copper foil, can be nonrecurring and elongated up to A = 300% [20].

### 3.2. Cyclic Mechanical Load

**Figure 9:**
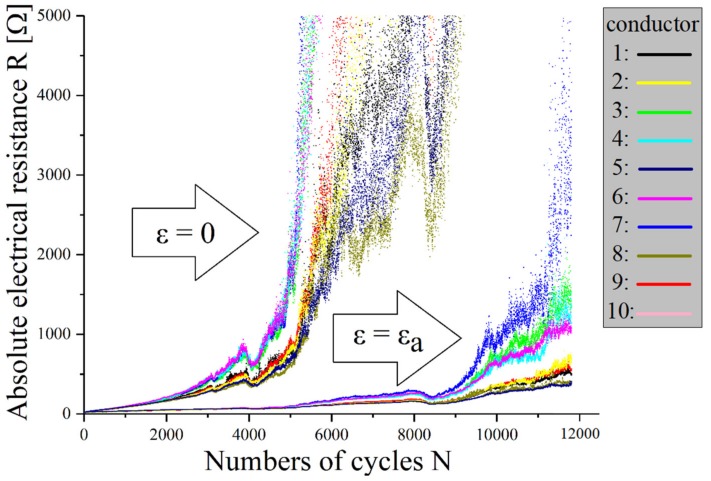
Electrical resistance changes of elastic textile/polymer composite-based stretchable electronics with conductors made of silver-plated knitted textiles under cyclic mechanical load at a strain amplitude of 22.1%. For definition of measurements, see Figure 6.

With cyclic mechanical loading of the samples, no classical conductor break was observed, but no or only a very late electrical failure occurred. Experiments demonstrate that the cyclic load at silver-plated fabrics causes a resistance increase up to 10,000 times the initial value, whereby significant differences between loaded and unloaded measurements can be seen. The improved conductance in loaded measurements could be due to the increased number of contact points due to cross-sectional contraction (see Figure 9).

Thus, the tests are evaluated on a specific application. A body conformable, textile-based electrode is used for the wearable measurement of vital parameters, for example for ECG or EMG measurements. Recent publications describe promising results for capacitively-coupled textile electrodes compared to skin contact dry electrodes [21,22]. High electrode impedance may increase noise, but this effect can be reduced by using appropriate (digital) signal processing techniques. In our example, therefore, an absolute electrical resistance increase including supply line of the textile electrode up to Rmax = 1 kΩ can be used as a failure criterion.

**Figure 10:**
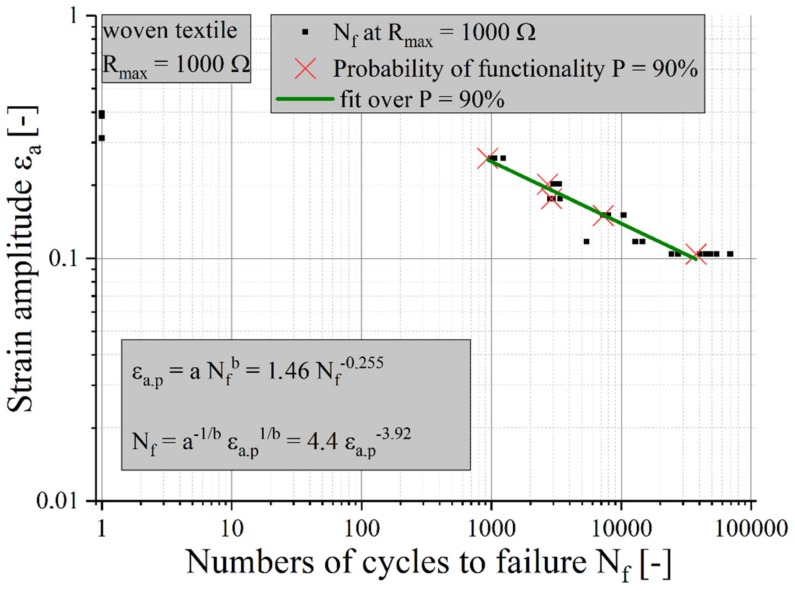
Life expectancy for elastic textile/polymer composite-based stretchable electronics with conductors made of silver-plated woven textiles under a cyclic mechanical load with R_max_ = 1 kΩ.

A statistical evaluation according to Rossow [23] allows one to determine a survival probability (90%) of the samples. According to Coffin [24] and Manson [25], an evaluation of the measurement results is possible (see Figure 10), so the life expectations of various stretchable circuit boards can be compared to each other.

**Figure 11:**
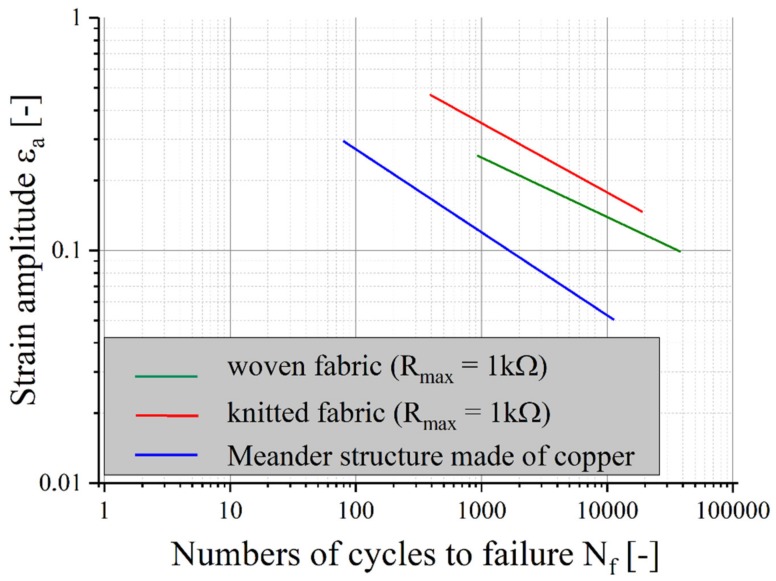
Life expectancy for elastic textile/polymer composites with conductors made of silver-plated woven or knitted textiles (R_max_ = 1 kΩ) compared with samples made of meandering copper tracks [26].

The results of the Coffin–Manson equations for samples with textile structures with the failure criterion of Rmax = 1 kΩ are shown in Figure 11. To compare the lifetime, the life expectancy of a sample with meandering copper tracks is added [26].

In the chosen application example, the life expectancy of samples with textile conductor materials is much higher than that of copper foil. The lifetime of the knitted structure is the highest among the samples tested. However, if another application requires less resistance, life expectancy will decrease for textile materials only. For applications where higher electrical resistance is sufficient, for example as electrodes, antennas or sensors, stretchable circuit boards with conductors made of textiles are very well suited.

### 3.3. Analysis of Failure Mechanisms

The microscopic analysis compares a heavily stressed sample with knitted conductor tracks (300,000 cycles at 25% strain amplitude) with an untested sample.

#### 3.3.1. Transversal Microsection

**Figure 12:**
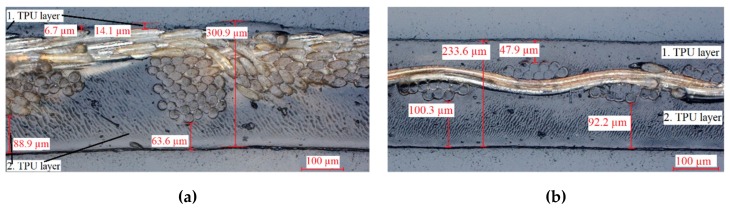
Micrographs of the elastic textile/polymer composites: (**a**) knitted conductive textile; (**b**) woven conductive textile.

In order to observe the internal structure of the untested samples, transversal microsections were made and analyzed with a visible light microscope (VLM) (see Figure 12).

Unlike the composite with conductive woven fabric, the knitted sample is not in the center of the TPU matrix and thus not in the neutral fiber. During the first lamination, the TPU layer melts and the knitted material is pressed into the TPU. In the second lamination step, TPU flows into the remaining gaps and bonds to the first TPU layer. However, the knit is pressed until the edge of the material compound. As a result, some polyamide threads are exposed, and they are no longer isolated and can react with the environment.

#### 3.3.2. Surface Analysis

**Figure 13:**
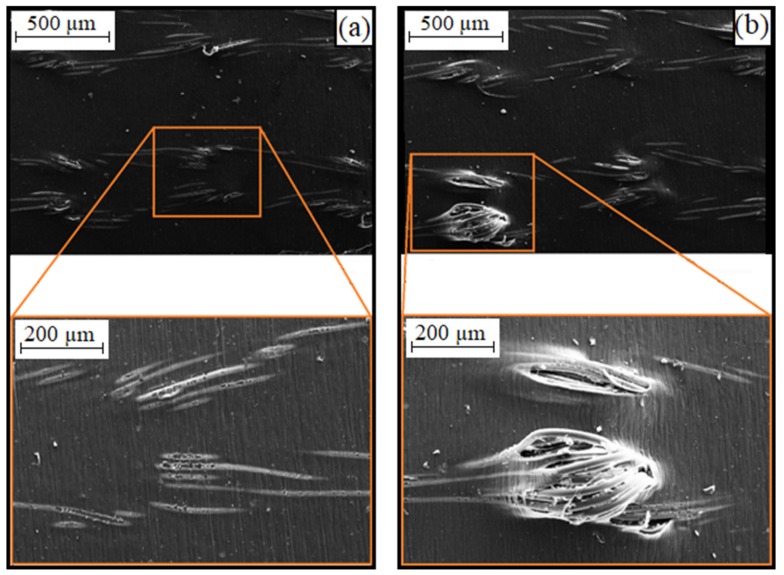
SEM images of samples with conductors made of knitted textiles: (**a**) untested; (**b**) loaded (300,000 cycles at a strain amplitude of 25%).

The exposed polyamide threads of the knit-based composite in the TPU layer can also be seen on the SEM images (see Figure 13, left).

**Figure 14:**
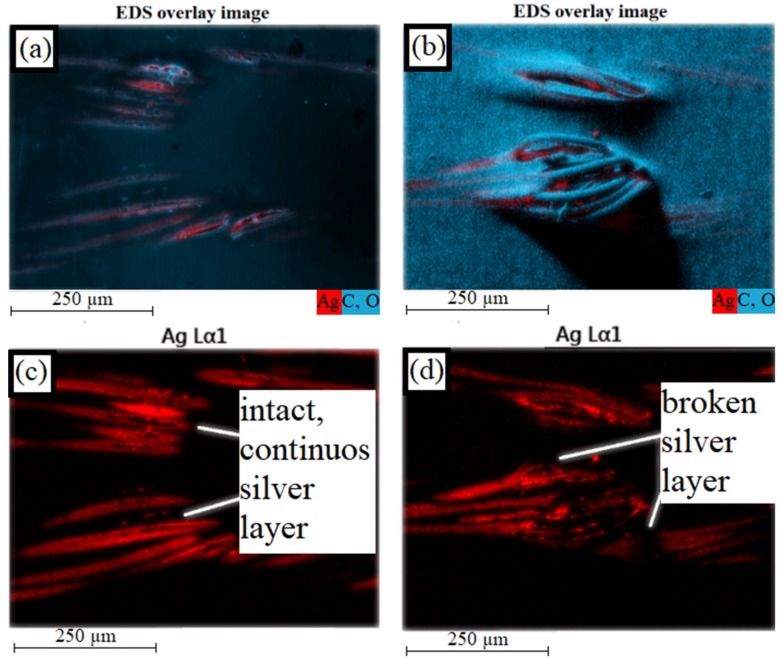
EDS overlay image of knitted sample (silver in red, polymer in blue): (**a**) untested; (**b**) loaded; EDS overlay image of knitted sample (only silver): (**c**) untested; (**d**) loaded.

Energy-dispersive X-ray spectroscopy shows that the silver layer on the polyamide filaments is still intact (see Figure 14, left). If the number of cycles increases, the defects in the TPU matrix become larger, so that the polyamide threads can move further out of the composite material (see Figure 13, right). Due to the repeated stretching and bending of the threads, the silver layer breaks (see Figure 14, right). This results in an increase in electrical resistance.

#### 3.3.3. Analysis of the Inner Structure

**Figure 15:**
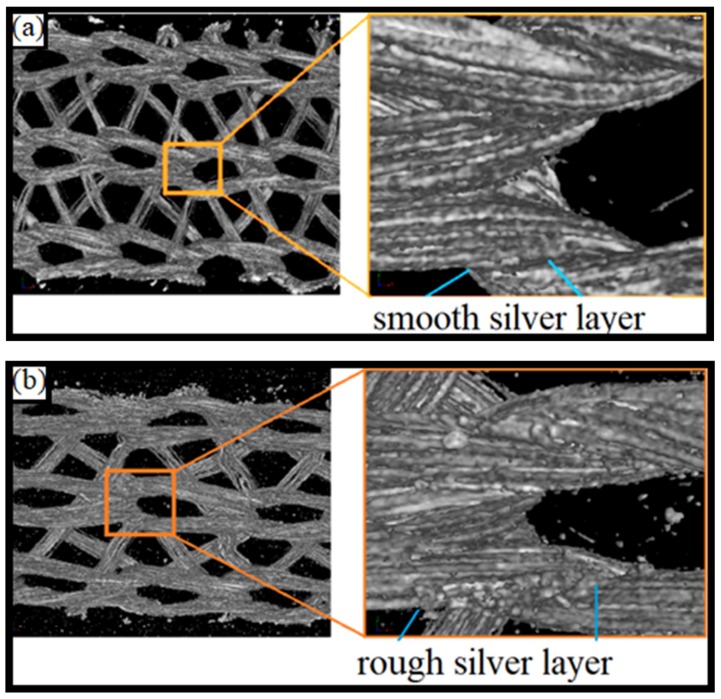
Micro CT as a 3D view of a sample with conductors of knitted textiles: (**a**) untested reference sample; (**b**) tested sample after 300,000 cycles at a strain amplitude of 25%.

In order to investigate the internal structure of the composite material, micro computed tomography (micro CT) is used. In the areas between the loops, even in the parts of their cross-connections, the untested fiber surfaces are still largely smooth. In the tested sample it can be seen that in the area of the loops, the previously smooth surfaces become rougher (see Figure 15).

**Figure 16:**
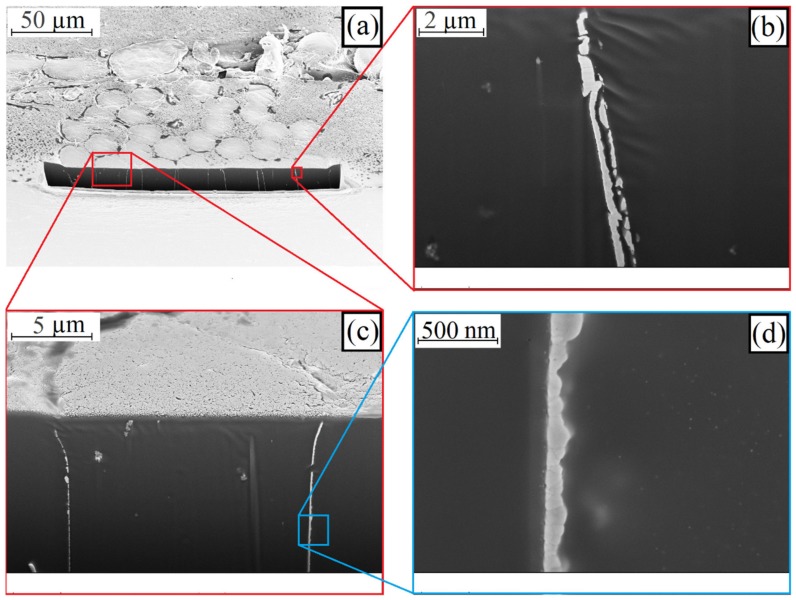
SEM image of a FIB cut on an untested sample with conductors made of knitted textiles at magnification of: (**a**) 500×; (**b**) 10,000× (**c**) 4000× and (**d**) 50,000×.

Knowing that the greatest damage to the silver layer occurred in the contact areas of the loops, transverse microsections of an untested and a tested sample were prepared and analyzed using a focused ion beam (FIB) and SEM in the contact area. Figure 16 shows the polyamide threads of the untested sample.

The silver nanoparticles on the filaments are sintered together to form a continuous, no uniform layer. This layer is around 50 nm to 200 nm thick (see Figure 16, bottom, right). The individual crystallites are still clearly visible. Complete sintering does not occur. The sintering takes place beyond the boundaries of a silver layer. If two silver-plated polyamide filaments are contacted to each other, the layers partially sinter together during lamination (see Figure 16, top, right). The gap between the layers shows that the FIB cut does not exactly pass through the contact plane at this point. The cut is either a little before or behind the contact plane.

**Figure 17:**
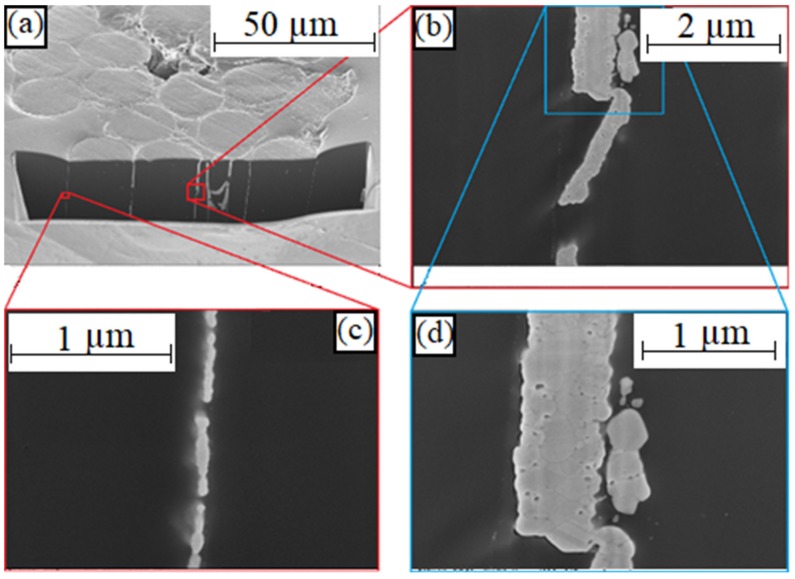
SEM image of a FIB cut on a tested sample with conductors made of knitted textiles at magnification of: (**a**) 1000×; (**b**) 20,000×; (**c**) 50,000× and (**d**) 40,000×.

The FIB cut on a tested sample (see Figure 17) shows a discontinuous silver layer on the polyamide filaments (see bottom, left). In the right images, several fibers meet so that the silver layer is thicker than that of a single fiber. Partially, whole silver-flakes dissolved and shifted within the material composite (see right).

## 4. Discussion

In this work, the mechanical and electrical properties of elastic textile/polymer composites for stretchable circuit boards under quasi-static or cyclic mechanical load were investigated on three conducting fabrics (knitted, woven, and nonwoven) embedded in a thermoplastic elastomer matrix.

It was observed that after lamination, the sheet resistances of all tested fabrics were reduced due to a sintering of silver nanoparticles and an increase in the contact points of the conductive fibers with each other. The silver-plated knit shows the best properties in the results of this investigation. It has the lowest initial and the least increase in sheet resistance under quasi-static mechanical load. In particular, the metallized knit fabric has the highest life expectancy under a cyclic mechanical load and thus exceeds the life expectancy of commercially available stretchable circuit boards based on thermoplastic polyurethane and meander-shaped conductor tracks made of copper foil.

With a cyclic mechanical tensile load of the textile–elastomer composite, no classical conductor cracks could be observed. Instead, the silver-plated polyamide fibers rub against each other at the loop of courses and wales intersections, so that over time the thin silver layer partially flakes off from the filament, thereby decreasing the electrical conductivity in the loop running direction. However, due to the presence of secondary filaments that transverse the loop running direction, which are subjected to lower linear mechanical load and friction, the electrical conductivity is maintained but decreases with mechanical load over time.

Due to the relatively high sheet resistances of conductive fabrics and the described electrical behavior under a mechanical load, an application, in particular, in the field of body-near textile sensors and electrodes is conceivable. Therefore, a life expectancy of a typical textile-integrated electrode with high mechanical strain as it occurs for example in knitted shirts was presented.

## 5. Conclusions

In the next step, the lamination of the conductive textiles into the elastomer matrix has to be optimized in order to prevent out-of-plane displacement and thus substantial damage to the surface metallization of the filaments. A further optimization of the mechanical load capacity is expected by a meandering design of the tracks, analogous to the currently used concepts for copper foil based stretchable conductor structures. Due to the mechanical failure behavior of the tested materials, no conductor breaks occur up to a high load, and we expect a good washing resistance of the textile/ elastomer-based circuit board. In conjunction with conductive textiles with a thicker surface metallization, the novel composite and manufacturing concept presented here can be used for most e-textile and smart clothing applications as well as multi-layer textile circuit boards for the realization of more complex textile-integrated electronic systems. Further fields of application are conceivable in the field of soft robotics. Pneumatically controlled, active morphing soft elastomer surfaces can be extended with conductive textiles to an electronic skin with sensor functions. 

## Figures and Tables

**Table 1:** Mechanical and electrical properties of the used materials [9,10,11,12].

Property	Unit	TPU	PA 6.6	Ag
melting temperature T_m_	[°C]	160	260	962
glass transition temperature T_G_	[°C]	−40	70	/
electrical conductivity (at 20 °C) κ	[Ωm]^−1^	10^−9^	10^−12^	61.4 × 10^6^
strain at break (at 20 °C) A	[%]	650	20	50
tensile strength (at 20 °C) R_m_	[MPa]	50	85	125–195

**Table 2:** Boundary conditions of the tensile test.

Parameter	Unit	Value
strain rate	[$\frac{\%}{min}$]	1
entire sample length	[mm]	100
test length l_0_	[mm]	31

**Table 3:** Parameters of the cyclic tests.

Parameter	Unit	Value
test frequency f	[Hz]	0.04
entire sample length	[mm]	200
test length l_0_	[mm]	100

**Table 4:** Mechanical and electrical properties of tested elastic textile/polymer composite-based stretchable circuit boards.

Property	Unit	Woven Textile	Knitted Textile	Nonwoven Textile
max. force F_max_	[N]	21–22.5	19–21	11–13
strain at break A	[%]	31–40	65–74	70–75
initial resistance R_0_	[Ω]	4.8–6.4	2.4–2.8	7.8–20.1
initial sheet resistance $R_{0,\;square}$	[mΩ/□]	120–160	60–70	195–502.5
resistance at break R_break_	[Ω]	11.5–16.4	7.4–8.9	61–100
max. resistance change $\Delta R_{max}=\frac{R_{break}}{R_{0}}$	[-]	2.5–3.5	2.6–3.7	5–8

## References

[1] Kallmayer C., Schaller F., Löher T., Haberland J., Kayatz F., Schult A. Optimized Thermoforming Process for Conformable Electronics. Proceedings of the 2018 13th International Congress Molded Interconnect Devices (MID).

[2] Wang C.F., Wang C.H., Huang Z.L., Xu S. (2018). Materials and Structures toward Soft Electronics. Adv. Mater..

[3] Steinmann W., Schwarz A., Jungbecker N., Gries T. (2014). Fibre-Table—Electrically Conductive Fibres.

[4] Linz T. (2011). Analysis of Failure Mechanisms of Machine Embroidered Electrical Contacts and Solutions for Improved Reliability. Ph.D. Thesis.

[5] Simon E.P. (2009). Analysis of Contact Resistance Change of Embroidered Interconnections. Bachelor’s Thesis.

[6] Breckenfelder C., Dils C., Seliger H.W. Electrical properties of metal coated polyamide yarns. Proceedings of the 4th International Forum on Applied Wearable Computing 2007.

[7] Tangsirinaruenart O., Stylios G. (2019). A Novel Textile Stitch-Based Strain Sensor for Wearable End Users. Materials.

[8] Isaia C., McNally D.S., McMaster S.A., Branson D.T. (2019). Effect of mechanical preconditioning on the electrical properties of knitted conductive textiles during cyclic loading. Text. Res. J..

[9] Eyerer P., Schüle P. (2019). Polymer Engineering 1.

[10] Hornbogen E., Warlimont H. (2016). Metalle—Struktur und Eigenschaften der Metalle und Legierungen.

[11] Product Information Platilon U. https://solutions.covestro.com/-/media/covestro/solution-center/brands/downloads/imported/1561582168.pdf.

[12] Elastollan—Material Properties. http://www.polyurethanes.basf.de/pu/solutions/elastollan/en/function/conversions:/publish/content/group/Arbeitsgebiete_und_Produkte/Thermoplastische_Spezialelastomere/Infomaterial/elastollan_material_uk.pdf.

[13] Mark J.E. (2011). Physical Properties of Polymers Handbook.

[14] Tobler-Rohr M.I. (2011). The supply chain of textiles. Chapter 2 Handbook of Sustainable Textile Production.

[15] Adams D., Alford T.L., Mayer J.W. (2008). Silver Metallization—Stability and Reliability.

[16] Von Krshiwoblozki M., Linz T., Neudeck A., Kallmayer C. (2013). Electronics in Textiles—Adhesive Bonding Technology for Reliably Embedding Electronic Modules into Textile Circuits. Adv. Sci. Technol..

[17] Holm R. (1967). Electric Contacts, Theory and Applications.

[18] Foerster P. (2010). Untersuchungen zu Eigenschaften von Nanosilberschichten auf Polyamidfasern. Student Research Project as Part of the Degree Programm Electrical Engineering.

[19] Born S. (2010). *Konzeptionierung* und *Realisierung* eines *Prüfstandes* zur *Qualifizierung* von *Dehnbaren Schaltungsträgern*. Bachelor’s Thesis.

[20] Vieroth R., Löher T., Seckel M., Dils C., Kallmayer C., Ostmann A., Reichl H. Stretchable Circuit Board Technology and Application. Proceedings of the 2009 International Symposium on Wearable Computers.

[21] Taelman J., Adriaensen T., van der Horst C., Linz T., Spaepen A. Textile Integrated Contactless EMG Sensing for Stress Analysis. Proceedings of the 2007 29th Annual International Conference of the IEEE Engineering in Medicine and Biology Society.

[22] Fuhrhop S., Lamparth S., Heuer S. A textile integrated long-term ECG monitor with capacitively coupled electrodes. Proceedings of the 2009 IEEE Biomedical Circuits and Systems Conference.

[23] Rossow E. (1968). Introduction to the Principles and Application of Sampling Plans by Attributes.

[24] Coffin L. (1954). A study of cyclic thermal stress on a ductile metal. Trans. Am. Soc. Mech. Eng..

[25] Manson S. (1954). Behaviour of Materials under Conditions of Thermal Stress.

[26] Grams A., Kuttler S., Löher T., Walter H., Wittler O., Lang K.-D. Lifetime modelling and geometry optimization of meander tracks in stretchable electronics. Proceedings of the 2018 19th International Conference on Thermal, Mechanical and Multi-Physics Simulation and Experiments in Microelectronics and Microsystems (EuroSimE).

